# The Evidence-Based Medicine Management of Endometriosis Should Be Updated for the Limitations of Trial Evidence, the Multivariability of Decisions, Collective Experience, Heuristics, and Bayesian Thinking

**DOI:** 10.3390/jcm14010248

**Published:** 2025-01-03

**Authors:** Philippe R. Koninckx, Anastasia Ussia, Assia Stepanian, Ertan Saridogan, Mario Malzoni, Charles E. Miller, Jörg Keckstein, Arnaud Wattiez, Geert Page, Jan Bosteels, Emmanuel Lesaffre, Leila Adamyan

**Affiliations:** 1 Departments of Obstetrics and Gynecology, Katholieke University Leuven, 3000 Leuven, Belgium; 2 Departments of Obstetrics and Gynecology, University of Oxford, Oxford OX1 2JD, UK; 3 Departments of Obstetrics and Gynecology, University Cattolica, del Sacro Cuore, 00168 Rome, Italy; 4 Departments of Obstetrics and Gynecology, Moscow State University, 119991 Moscow, Russia; 5 Gruppo Italo Belga, Villa del Rosrio, 00191 Rome, Italy; 6 Academia of Women’s Health and Endoscopic Surgery, Atlanta, GA 30328, USA; 7 Elizabeth Garrett Anderson Institute for Women’s Health, University College London, London WC1E 6 AU, UK; 8 Malzoni Research Hospital, 83100 Avellino, Italy; 9 Department of Clinical Sciences, Rosalind Franklin University of Medicine and Science, Chicago, IL 60064, USA; 10 Department of Minimally Invasive Gynecologic Surgery, Advocate Lutheran General Hospital, Park Ridge, IL 60068, USA; 11 Endometriosis Centre, Dres. Keckstein, 9500 Villach, Austria; 12 Faculty of Medicine, University Ulm, 89081 Ulm, Germany; 13 Departments of Obstetrics and Gynecology, Faculty of Medicine, Latifa Hospital, Dubai 9115, United Arab Emirates; 14 Departments of Obstetrics and Gynecology, University of Strasbourg, 67081 Strasbourg, France; 15 Coordinator Clinical Guidance Project VVOG, 9100 Sint-Niklaas, Belgium; 16 Departments of Obstetrics and Gynecology, AZ Imelda, 2820 Bonheiden, Belgium; 17 Department of Human Structure and Repair, University of Ghent, 9000 Ghent, Belgium; 18 I-Biostat, Faculty of Medicine, KULeuven, 3000 Leuven, Belgium; 19 Department of Operative Gynecology, Federal State Budget Institution V. I. Kulakov Research Centre for Obstetrics, Gynecology, and Perinatology, Ministry of Health of the Russian Federation, 117997 Moscow, Russia; 20 Department of Reproductive Medicine and Surgery, Moscow State University of Medicine and Dentistry, 127473 Moscow, Russia

**Keywords:** endometriosis, laparoscopic surgery, evidence-based medicine, black swan, heuristics, experience-based medicine

## Abstract

**Background/Objectives**: The diagnosis and treatment of endometriosis should be based on the best available evidence. Emphasising the risk of bias, the pyramid of evidence has the double-blind, randomised controlled trial and its meta-analyses on top. After the grading of all evidence by a group of experts, clinical guidelines are formulated using well-defined rules. Unfortunately, the impact of evidence-based medicine (EBM) on the management of endometriosis has been limited and, possibly, occasionally harmful. **Methods**: For this research, the inherent problems of diagnosis and treatment were discussed by a working group of endometriosis and EBM specialists, and the relevant literature was reviewed. **Results**: Most clinical decisions are multivariable, but randomized controlled trials (RCTs) cannot handle multivariability because adopting a factorial design would require prohibitively large cohorts and create randomization problems. Single-factor RCTs represent a simplification of the clinical reality. Heuristics and intuition are both important for training and decision-making in surgery; experience, Bayesian thinking, and learning from the past are seldom considered. Black swan events or severe complications and accidents are marginally discussed in EBM since trial evidence is limited for rare medical events. **Conclusions**: The limitations of EBM for managing endometriosis and the complementarity of multivariability, heuristics, Bayesian thinking, and experience should be recognized. Especially in surgery, the value of training and heuristics, as well as the importance of documenting the collective experience and of the prevention of complications, are fundamental. These additions to EBM and guidelines will be useful in changing the Wild West mentality of surgery resulting from the limited scope of EBM data because of the inherent multivariability, combined with the low number of similar interventions.

## 1. Introduction

Evidence-based medicine (EBM) was introduced as a way to use evidence as a new teaching approach [1]; it was subsequently defined as “the conscientious, explicit and judicious use of current best evidence in making decisions about the care of individual patients, and thus integrates individual clinical expertise with the best available evidence from research” [2]. Because of the need for (frequentist) statistical analysis to judge evidence, EBM took off after computing power facilitated the necessary calculations. Emphasizing the need to avoid the many well-known cognitive biases in medical decision-making [3,4], the pyramid of evidence [5] was topped with the double-blind, randomized controlled trial (RCT), which is still, today, the gold standard of research [6] since this theoretically avoids inclusion, observer, and patient bias. Later, meta-analysis and systematic reviews were added on top of the pyramid. When the limitations of RCTs and the need to incorporate non-perfect trials and experience were realized, the quality of evidence was graded by a group of experts and translated into clinical guidelines, using well-defined rules for grading [6] and guideline development [7].

Notwithstanding important achievements, the clinical impact of EBM has remained limited [8,9], especially in surgery [10] and in endometriosis management [11]. It is even unclear whether EBM has improved surgical outcomes. This limited impact can be explained by difficulties in organizing surgical trials, in the statistical interpretation of results, the *p*-value fallacy [12], the inappropriate use of post hoc subgroup analysis [13], the inappropriate use of frequentist statistics [14], and the indirect evidence that most published research findings must be wrong [15], requiring the de-implementation of some healthcare practices [16]. Bayesian statistics [17], learning from the past, the value of clinical experience, heuristics, and intuition are still poorly incorporated [18]. This explains the limited recognition of the multivariability of clinical decisions and the occasionally subjective, although well-defined [19], grading of evidence by a panel of experts with different backgrounds and fields of expertise. As a result, the guidelines formulated by the European Board and College of Obstetrics and Gynaecology, the American Society of Reproductive Medicine, the European Society of Human Reproduction, and others are slightly different, with little clinical guidance used for diagnosis, medical treatment, and surgery [11].

Some of these observations on EBM will be discussed below, with endometriosis management as an example. Endometriosis is a frequently diagnosed disease occurring in over 10% of women, with a clinical presentation varying from small, subtle lesions to cystic ovarian endometriosis and severe, deep lesions. Endometriosis is a major cause of pelvic pain and infertility, with an estimated 7-year delay in diagnosis. Endometriosis management remains debated because of specific problems. Experimentation is limited without an appropriate animal model. The non-invasive, accurate diagnosis of endometriosis is difficult, limiting data to those patients with sufficient symptoms to be recommended for laparoscopy. Medical treatment cannot be blinded when the patient recognizes the active drug, and the low number and complexity of surgical interventions are prohibitive for RCTs.

## 2. Materials and Methods

A working party was formed, consisting of specialists in endometriosis, mainly surgeons with a cumulative experience of over 50,000 treatments of women with endometriosis, as described previously [20], along with specialists in EBM and Bayesian statistics. Our aim was to identify why the impact of EBM in the management of endometriosis has remained limited, to review the literature describing the limitations of EBM as implemented today, and to suggest how to bridge the gap between EBM and clinical medicine, especially regarding surgery.

## 3. Results

### 3.1. The Limitations of RCTs: Rare Events and Multi-Variability

The RCT is not suited to rare events, such as the complications of surgery, since obtaining 30 cases to study a 1% event would require the inclusion of 3000 patients in the control group alone.

Another problem that has been poorly addressed is incorporating multivariability into a randomized controlled trial. To investigate two factors, A and B, and their eventual interaction, a factorial design [21,22] with four groups (control or A^−^B^−^, A^+^B^−^, A^−^B^+^, and A^+^B^+^) is required. The simultaneous investigation of three factors requires 8 groups, and if each factor has three levels (0, +, ++), three factors will result in 27 groups and five levels of five factors in 3125 groups (groups = levels^number of factors^) [23]. A factorial design with the same number of patients evaluates the effect of each factor separately, along with their interaction, almost without a loss in statistical power. However, notwithstanding these advantages of investigating interaction, and although requiring only half the number of patients compared to two one-factor trials, the randomization problems of multivariate trials and the high number of patients that must be included are prohibitive. Also, independent, dependent, associated, and continuous factors should be considered. For example, age, which is strongly associated with multimorbidity, is difficult to dissociate from the associated diseases [24].

**Figure 1:**
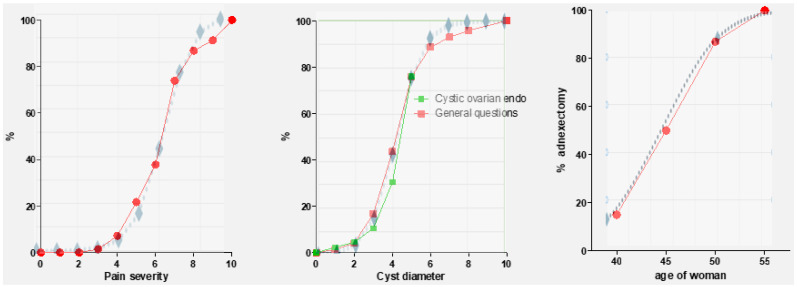
Indications for surgery are multivariate, with at least the severity of pain, the diameter of the cyst, the age of the woman, and other factors such as CA125 and fertility. The red and green lines show the percentage of gynecologists performing surgery when pain severity or the cyst’s diameter are the only symptoms. The third graph illustrates the percentage of adnexectomies at different ages for a 4 cm endometrioma and a CA125 of 50 IU (reprinted with permission from Ref. [26]). The blue dotted lines illustrate the goodness of fit when the relationship is calculated as a sigmoidal relationship between pain 1/(1 + e^−1.2(P−6.2)^), diameter 1/(1 + e^−1.4(D−4.2)^), and age 1/(1 + e^−4(A−4.5)^), respectively.

We risk forgetting that randomization cannot solve the inherent problems of a one-factor trial. These trials are a simplification of the clinical reality since diagnosis and treatment decisions are multifactorial, often with strong interactions. The diagnosis of endometriosis is a sequential process of Bayesian updating and elimination, by which process the probability of each of the many potential diagnoses is progressively updated when new data become available. “It is a process of progressively reducing uncertainty about a diagnosis by new information to reach a conclusion with a sufficiently high probability to guide therapy” [18]. These probabilities begin by taking into account the patient’s age and antecedents and their heredity and symptoms, then these probabilities are progressively updated with the results of clinical exams, imaging, and other exams, as previously illustrated effectively for Kartagener syndrome [25]. Also, treatment decisions are multifactorial. An example is the indications for surgery in cystic ovarian endometriosis. This is a clinical decision based on a combination of the severity of pain, cyst diameter, the imaging aspect, the age of the female patient (Figure 1), and the patient’s expectations [26].

**Figure 2:**
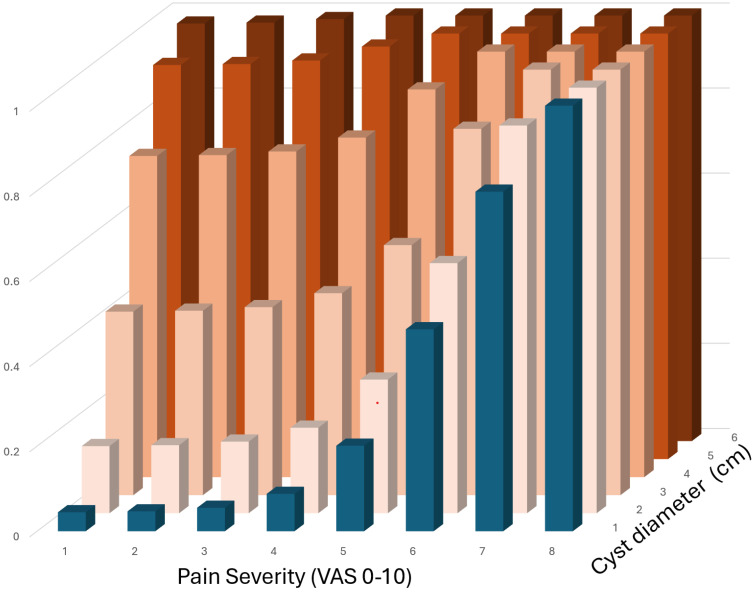
The percentage og gynaecologists performing surgery was calculated for any combination of pain and cyst diameter assuming a sigmoid relationship and no interaction. Using the sigmoidal relationship between the percentage of gynecologists performing surgery due to the severity of pain (blue bars representing data of left graph of Figure 1; percentage performing surgery = 1/(1 + e^−1.2(P−6.2)^) and the cyst diameter (left of the graph—intensity of red colours; percentage performing surgery = 1/(1 + e^−1.4(D−4.2)^), thet percentage gyneclogists performing surgery was calculate for any combination, of pain and cyst diameter assuming no interaction.

The percentage of surgeons performing surgery shows a sigmoidal relationship with the severity of pain and cyst diameter pain, as shown in the overlay in Figure 1. Using these sigmoidal relationships, a composite graph was constructed by adding the probabilities of surgeons performing surgery to relieve pain and due to the diameter of the cyst by adding the percentages for pain and cyst diameter (Figure 2). However, the interaction between these factors remains unknown, i.e., whether the percentage can be added to, reinforced, or weakened at certain points. This illustrates the complexity of the relationship when several factors are involved and shows the limitation of one-factor trials when the decisions are multivariate.

Also, surgical interventions and their outcome depend on many factors, such as the indications’ complexity, the intervention’s type and severity, and the surgeon’s skills. The clinical impact of a one-factor RCT will, thus, be limited, whereas a multivariate trial is close to impossible because of randomization issues and the relatively small number of comparable interventions by each surgeon. We should realize that the results of a case series arise from a specific combination of indications, the severity of the disease, the strategic and tactical choices made during surgery, the individual surgeon’s skills, their experience, and the local setting. This highlights the gap between the diagnostic and treatment decisions taken daily by the clinician and the limited evidence available.

### 3.2. Heuristics and Intuition

Heuristics are an evolutionary adaptation [27] that simplifies problem-solving using mental shortcuts, permitting quick decisions or judgments in complex situations without processing all the relevant information [28,29]. When you see a person with a hood in a dark alley and decide to walk past a little faster, your brain has probably used a heuristic to evaluate the situation as being potentially dangerous. Clinicians use heuristics to make quick decisions based on experience and intuition rather than detailed analysis [30]. Skills development, such as surgery, is another form of heuristics.

Heuristics are important when speed matters and the cognitive load is high, a situation experienced by professionals in high-stress, high-uncertainty environments, such as soldiers and surgeons [30]. Heuristics develop from experience and training until they become almost automatic, as demonstrated in sports training and by the learning curves in surgery [31]. Other examples are firefighters who develop an intuitive sense of when a burning building might collapse, and differences in the eye movements of gunfighters and police officers with and without experience [32], illustrating their ability to focus on the more critical dangers. Also, eye movements during image analysis differ between the experienced and the less experienced user, with the former showing greater accuracy and increased speed [33,34,35,36].

Heuristics in surgery are well documented. Surgery requires ’tricks of the trade’ in both manual and perceptual skills [37]. Heuristics are ’rules of thumb’ that experts learn through trial and error. Technical surgical heuristics are manual and perceptual, with haptic feedback and cognitive skills [37], similar to those demonstrated in many areas of work, sport, and the arts. Expert surgeons have a mental library of images and patterns, permitting them to perform complex tasks almost subconsciously on automatic pilot without distinguishing the components of complex movements, a process called chunking. These heuristics permit them to focus on the critical aspects, recognize and adjust to abnormal circumstances, and recognize and correct small mistakes.

The precision of heuristics risks being less reliable [38] since it is less conscious, with many cognitive biases. For example, a surgeon learns from encountering complications, but discussing one’s shortcomings is not enjoyable, and acknowledging complications might be strategically detrimental in a highly competitive environment. Many well-known cognitive biases have been described for neurosurgery, such as the self-serving bias, the actor–observer effect, heuristic biases in interpreting probabilistic events, the representativity bias, the availability bias, emotional avoidance and denial, limitations of attention, or dual systems theory and errors of memory [39]. Without discussing the many cognitive biases, we should realize that they may hamper the realistic understanding by the surgeon of his surgical skills and his daily risk-taking [40]. An important difference between heuristics and intuition is that intuition is more subconscious and, thus, more vulnerable to emotional influences and cognitive biases.

Heuristics in medicine and surgery deserve investigation to understand how they develop from experience and training to the stage of automation. We know that acquiring motor skills involves neuroplasticity and requires sleep to consolidate learned skills [41]. This explains why acquired skills are cumulative and are retained for longer periods [42,43]. It is also important that our conscious awareness of these shortcuts decreases while our skills become increasingly automated. This decreased conscious awareness also explains why the very skilled can be poor teachers. It also emphasizes the need for a coach or trainer to teach and improve heuristics and prevent ‘bad habits’. This also illustrates the importance of debriefing, analyzing movements in slow motion, and identifying near-misses, as is performed when training fighter pilots (R. Mashiach, Congress presentation). As is well known in many sports, a team is more than the sum of the individuals. Team building is another form of heuristic development, emphasizing the importance of the surgeon and the assistants acting as a team.

Heuristics and EBM are not contradictory, and heuristics may align closely with EBM. However, biases must be recognized because of the risk of cognitive biases, up to cognitive dissonance [44]. For example, realizing the risk of availability bias should incentivize the clinician to look up current evidence, rather than relying solely on memory.

### 3.3. Bayesian Thinking or Learning from the Past

Bayesian thinking formally updates past data when new data becomes available. The weather forecast uses past information on weather patterns in a given location to predict the weather or the probability of rain in the future. Since the prediction for tomorrow is more accurate than for next week, the predictions are updated each day with new information from that day. Humans think Bayesian and the results of cooking will be used for the next cooking. Also, medicine thinks Bayesian [18] when narrowing down the probabilities of a diagnosis when the results of new exams become available [25]. A surgeon [45] will update his procedure after a complication occurs. Bayesian thinking is also more formally aware of the many factors influencing a decision. Bayesian statistics is a similar process, using the accuracy and distribution of all the information we have (the prior) to calculate the new (the posterior) probability when new data become available, emphasizing uncertainty.

Experience evolves with each new diagnosis or treatment, from a new publication to learning the experiences of others. These experiences can be used to update our past thinking formally, but they also translate into intuition and heuristics, making the personal or individual experience prone to cognitive biases. Therefore, personal experience ranks low in the pyramid of evidence as a personal opinion. However, when many of us have similar experiences, this personal experience becomes a collective experience, with less risk of cognitive bias. Using the experience of managing the treatment of more than 50,000 women with endometriosis, we defined a collective experience as a similar experience by over 80% of gynecologists, and we defined a similar experience as one with more than 75% agreement with the VAS scale rating of an item [26]. The agreement was striking regarding many aspects of endometriosis management. Without discussing the evidence value of these documented collective experiences, they could be used to update or extrapolate prior EBM data obtained from a population sample into posterior probabilities in the entire population. Collective experience might also be useful in surgery to expand the limited EBM data to the many aspects of treatment without trial evidence [26,46].

Bayesian thinking is conditional thinking, which is important for many decisions in diagnosis and treatment. An example is the clinical value of imaging, either ultrasound or MRI, in diagnosing deep endometriosis. The clinician wants to know the probability that a woman with a positive test has the disease and that of the risk of missing the presence of disease when the test is negative. These are the predictive values of a test. Bayesian thinking explains that a positive test result only updates the known probability. A positive test with 90% sensitivity and specificity will update the probability of the 1% prevalence of a disease to an 11% probability of having that disease. Similarly, the excellent sensitivities and specificities, at around 90% for the imaging of deep endometriosis, will update the 2% to 3% prevalence of deep endometriosis to some 50% probability [47]. This also explains why PPVs decrease sharply when prevalences are less than 10% [48] and that PPVs are much higher in referral centers, with more than 10% prevalence because of referral bias. This imaging example also permits us to illustrate other biases when interpreting test results. Since most ultrasonographers take pain symptoms and elicited pain during the exam into account, it is difficult to estimate the added value of imaging, given that the pain symptoms were known already. We also should consider that imaging accuracy is artificially improved by not considering that the accuracy will likely decrease when the volume is less and that our data are limited to women undergoing a diagnostic laparoscopy. The results are, moreover, location- and operator-dependent. The clinical value of imaging thus should describe the added value of imaging given the symptoms and clinical exam, given the local prevalence of the disease, given the accuracy for this volume and given the operator skills. This is what most clinicians consider before deciding to perform surgery. A conscious realization of this complex conditional thinking by the surgeon may explain some discussions with ultrasonographers when considering only the accuracy of imaging. Another example is the recognition of early warning signs during patient recovery after surgery.

The absence of multivariate thinking is also reflected in elaborate decision trees depicting sequential univariate decisions, such as those published for cystic ovarian endometriosis [49]. Instead of these complex diagrams describing clinical multivariability as sequential decisions, it would be preferable to list the many variables involved in each decision and the probabilities of each alternative possibility, including the interactions.

### 3.4. Black Swans

Black swans are unpredictable events that have a huge effect and can only be explained in hindsight [50]. Although well-known in economics, they are less commonly discussed in medicine, especially in EBM, since they are generally rare and unpredictable without trial evidence. However, many events in medicine can be considered medical black swans, such as the plague in the Middle Ages, the syphilis imported from South America by Christopher Columbus, and COVID-19 more recently. Less widely recognized as black swans are discoveries, such as that of bacteria by Pasteur, the double helix of DNA, and some severe complications of surgery. Clinicians consider black swans by ranking their diagnoses and treatment, as well as by the harm they may cause, such as the risk of complications or missing a diagnosis of cancer [51]. We fail to prepare and train for life-threatening incidents that can and will occur, albeit only once in a lifetime. For example, trocars with luer locks that limit insufflation to 10 l/min are widely used, although we know that a large vascular accident often requires continuous aspiration, removing 20 L/min and resulting in a rapid loss of pneumoperitoneum [52].

The narrative fallacy of ordering and telling unrelated events as a story leads to inaccurate conclusions about cause-and-effect relationships [53] and is considered a subtle black swan heuristic. Although many complications are random events, we often explain afterwards how they happened and how they can be prevented. Guideline narratives risk containing black swans, and most practitioners will not assess the evidence [54]. Also, the pathology of endometriosis risks contains narrative fallacies when describing logical but not observed intermediary steps between images. The story of progressive differentiation to ’endometrium-like’ cells could be a narrative fallacy, obscuring the reality of stepwise changes after genetic or epigenetic incidents [55].

Black swan events continue to happen. The unexpected observation of a biphasic relapse pattern of breast cancer, caused by systemic inflammation after surgery, has the potential to become an important and unexpected Kuhnian paradigm shift [56] to prevent recurrence with anti-inflammatory drugs.

### 3.5. The Wild West Mentality in Surgery

The rare, solid EBM data and rules [57] risk a ’Wild West’ mentality in surgery [58,59,60,61]. The surgeon could consider as permissible those actions not demonstrated to be wrong, feeling entitled to act in what he considers to be the most appropriate way based on his personal experience and knowledge from education, the literature, or meetings. Equally important is that without video registration [62], few people can judge which decisions are taken regarding the individual patient. Although live surgery is important when transparent and discussed, it also offers a glimpse of this Wild West mentality, with different surgeons demonstrating and explaining their techniques and preferences. Examples of this technical variability without solid data are the direct first trocar insertion versus the use of Verres needle and insufflation to open insertion, the use of various energy sources such as electrosurgery or ultrasonic energy or lasers, different energy settings, excisional deep endometriosis surgery following the borders of the lesion versus the extensive prior dissection of all spaces, cystic ovarian endometriosis treated by excision, or superficial destruction or alcoholization, describing the largest diameter or the volume of deep endometriosis lesions, bowel resections for nodules of 2–3 mL, varying from a few percent to over 80%, and many other differences such as bowel preparations, ileostomies, drains, and the management of complications.

New surgical treatments are often adopted without adequate supporting evidence of their efficacy and safety [57,63,64]. The introduction of new techniques or new materials is often a personal choice based on understanding and experience [64]. Innovations aim to improve standard surgery, but few innovations have been designed as prospective trials, and failures risk not being reported. The many examples of introducing new techniques without proven superiority vary from the use of meshes to robotic surgery and the introduction of laparoscopic surgery. Even the basic aspects, such as knot sequences and the loop and knot security of suturing, were poorly investigated until recently [65,66,67].

An update of the limited EBM trial data, with collective experience data spanning all aspects of surgery, could be important to reduce the feeling of freedom and prevent this Wild West mentality.

## 4. Discussion

**Figure 3:**
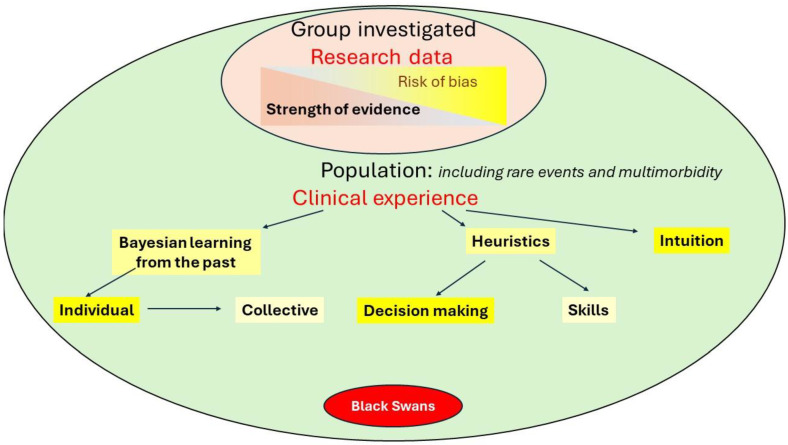
Endometriosis management is based on the evidence sourced from research data on the groups of women investigated (small rose-colored ellipse); this should be updated with clinical experience in the entire population (large green ellipse), including rare events and multimorbidity. Clinical experience comprises conscious Bayesian learning from the past, either individually or collectively, as well as heuristics in decision-making and skills and the less conscious form of intuition. The risk of bias is indicated in yellow. However, decisions should consider not only the evidence but also the risk of causing harm, as indicated by black swans, which present as random and unpredictable complications.

The management of endometriosis should be based on the best available evidence. This is part of the definition of EBM, but it can also be ambiguous. The ’best available evidence’ varies from the pyramid of evidence emphasizing the design of a trial, the absence of bias, and the correct statistical inference, to the interpretation of all other evidence as being judged and graded by consensus, obtained from a group of experts with variable experience, including some with little clinical experience. Frequentist statistical inference cannot prove a hypothesis, which is known as the *p*-value fallacy [12,14], but this remains a frequent mistake in medicine. It seems wise that the interpretation of trial results, statistical inference, and surgery is performed by those with experience in these different aspects (Figure 3). For example, we cannot expect every clinician to be aware of Simpson’s paradox [68,69,70,71] when analyzing data.

The inherent limitations of RCTs should be realized. One-factor trials are a simplification of clinical reality, ignoring the multivariability of most decisions and the interaction between factors. Trials will not reflect unpredictable and rare events, such as some complications, which should be recognized as random events and black swans. We should be prepared for and trained to deal with them when they occur, rather than looking for an explanation and prevention. It should be realized that multidisciplinarity adds to the multivariability of decision-making, and that multicenter trials unavoidably add many variables. The inherent limitations of apparently well-performed RCTs in surgical oncology [72,73,74] explain that the conclusions remain debated and variably implemented.

Experience, heuristics, and intuition and the associated cognitive biases are complex, but they deserve investigation and integration into EBM. Understanding the heuristics of training and learning curves seems important to improve the teaching of skills, lesion recognition, and decisions. This might improve the recognition of endometriosis and all aspects of surgery, such as hand–eye coordination, dissection, energy use, stitching, and knot tying. Results might eventually permit the early differentiation of trainees [75] since not all of us will learn how to return a tennis ball reaching us at 200 km/h. The many cognitive biases of heuristics need recognition and investigation, as suggested by the systematic debriefings after surgery [76,77,78] and those of Air Force pilots (Mashiach R, MIT TED Talk/personal communication), emphasizing honesty when reporting near-misses. Heuristics are important for diagnosis, although it is more difficult to distinguish between the formal Bayesian sequential updating of probabilities and heuristics and intuition. Documented collective clinical experience [26] deserves investigation since it can avoid the cognitive biases of personal clinical experience.

Clinicians think and act in a Bayesian manner, but clinicians should be taught to learn more consciously how to update clinical experience and the multivariability of decision-making, and to understand the Pareto distribution of most complications, with 80% being caused by 20% of surgeons. We also need to understand narrative fallacies with a risk of becoming black swans.

Patient-oriented medicine [79] is the third dimension of medical decision-making, along with evidence-based medicine and its experience-based updates. The goal of patient-oriented medicine is to improve patient satisfaction with diagnosis and treatment [80,81,82]; this can be achieved by a respectful, culturally sensitive approach and competent care. However, patient-oriented medicine also emphasizes the shared decision made when the physician explains in detail the pros and cons of all the available treatments, not only those that he is able to offer [83]. The emphasis on psychology [84], patient involvement [85], patient-centered outcomes [85], and viewing experience as the art of interacting with the patient instead of considering experience as an update of EBM has previously caused confusion [86].

Also important for understanding medical decision-making is the sigmoidal relationship between the severity of pain or the diameter of an endometrioma and the indications for surgery, as shown in Figure 1. Although a decision can be yes or no, the sigmoidal shape starts with a hard no, followed by probably no, unclear, probably yes, and finally, a hard yes. This reflects the individual variability in judgment. It is most important to realize that the narrow limits of the steeply ascending part of the curve reflect the narrow limits between probably no, uncertainty, and probably yes. This is similar to confidence limits or the degree of uncertainty seen in evidence-based data. For patient-oriented decisions, it can be argued that these are important for the areas of uncertainty, but are much less important when data or clinical experience indicate a hard no or yes.

## 5. Conclusions

EBM should be considered prior data to be updated by heuristics and collective experience. We must understand and train the heuristics and biases of pattern recognition, manuality, and the early recognition of complications. A Bayesian awareness of the probabilities of each diagnosis and each decision during surgery or management includes learning from each other and from other disciplines, the regular review of surgical outcomes, and an emphasis on learning from mistakes and near-misses. These updated EMB guidelines might reduce the Wild West mentality of surgery by adding guidelines for the many aspects currently without trial evidence. Patient-oriented decisions are important, especially for areas with an uncertainty of evidence or experience-based conclusions.

## Data Availability

Not applicable.
